# Analysis of Predictors of Breast Cancer Screening among Japanese Women Using Nationally Representative Survey Data, 2001–2013

**DOI:** 10.31557/APJCP.2021.22.1.171

**Published:** 2021-01

**Authors:** Tasuku Okui

**Affiliations:** *Medical Information Center, Kyushu University Hospital, Fukuoka City, Japan. *

**Keywords:** Breast cancer, Cancer screening, Cross-sectional studies, Japan, Socioeconomic factor

## Abstract

**Objective:**

Predictors of participation in breast cancer screening in recent years or the trend of participation rate by predictors over the years has not been investigated in Japan. In this study, we investigated predictors for participation in breast cancer screening and analyzed the trend of participation rate depending on the predictors using nationally representative survey data in Japan.

**Methods:**

The data of “Comprehensive Survey of Living Conditions” in Japan from 2001 to 2013 were used. Participation in breast cancer screening was used as an outcome. Next, as explanatory variables, we used age group, marital status, living arrangements, educational level, household income, employment status, smoking status, regular outpatient visit status, and self-rated health status. Then, the participation rate for breast cancer screening was calculated for each of the factors over the years. In addition, multivariate logistic regression analysis was conducted to analyze the association between each factor and the participation rate using data from 2010 and 2013.

**Results:**

We found that non-married women, women with lower educational level, women with low household income, self-employed or unemployed women, smokers, and women with low self-rated health status were significantly less likely to participate in breast cancer screening. Conversely, the participation rate increased for all predictor groups from 2001 to 2013, and the increase in the participation rate for never-married women was particularly evident compared with the other marital statuses. However, significant differences in the participation rate for breast cancer screening existed depending on marital status, household income, employment status, and smoking status throughout the analyzed years.

**Conclusion:**

Our findings suggest that further recommendations for breast cancer screening are particularly needed among women of low socioeconomic status and those who are self-employed or unemployed to increase the participation rate in Japan.

## Introduction

Breast cancer is a representative type of cancer. According to the Patient Survey and the Vital Statistics in Japan, the estimated number of patients with breast cancer increased from 259.1 to 359.5 per 100,000 women between 1999 and 2017 (Ministry of Health, Labour and Welfare of Japan, 2020). In addition, the mortality rate for breast cancer increased from 13.9 to 23.0 per 100,000 individuals during the same period (Ministry of Health, Labour and Welfare of Japan, 2020). Also, although age-standardized mortality rates of representative cancer types decreased in this period, that for breast cancer increased (Okui, 2020). Identifying causes of the increase in breast cancer and taking preventive measures in Japan is important. Breast cancer screening is an important measure for detecting patients and starting medical care. In other countries, although some controversies exist (Miles et al., 2011), the introduction of breast cancer screening is said to have contributed to a decrease in breast cancer mortality (Otto et al., 2003; Kalager et al., 2010). The participation rate of breast cancer screening in Japan is known to be relatively low compared with Western countries (Sano et al., 2017), and an increase in the rate of participation is needed for early treatment of breast cancer.

One factor for the low participation rate for breast cancer screening is low income women, based on data from 2001 (Fukuda et al., 2005). Although the previous study investigated the association between socioeconomic factors and breast cancer screening rate, the data analyzed were from 2001; thus, the association in recent years remains uncertain. Although the participation rate for the screening of other types of cancer was investigated using recent data (Kaso et al., 2019; Maeda et al., 2020), the predictors for breast cancer screening were not investigated. Also, although there might exist some similarities between predictors for breast and cervical cancer screening, a previous study for cervical cancer did not investigate the predictors for age groups of over 40 years old, which are the main target age for breast cancer screening (Kaso et al., 2019). In addition, the previous study on breast cancer screening did not use predictors such as educational level, smoking status, and company size, which are known to be associated with participation rate of other types of cancer screening in recent years (Kaso et al., 2019; Maeda et al., 2020). In other counties, research demonstrated that educational level was also associated with the rate of participation (Damiani et al., 2015; Akinyemiju et al., 2016). A possibility exists that the association between income and participation rate was confounded by other factors in the previous study, and conducting an analysis using more diverse factors and seeking a method to encourage consultation screening to groups with low levels of participation is important. Furthermore, the trend of the participation rate for breast cancer screening by predictors over years was not revealed in a previous study in Japan. Whether or not the disparity among socioeconomic factors, including household income, increased over the years is not uncertain. Therefore, in the current study, we investigated predictors for participation in breast cancer screening. Also, we analyzed the trend of the participation rate depending on the predictors using nationally representative survey data in Japan.

## Materials and Methods

*Data*

The statistical results obtained in this study were made and analyzed by the author using the anonymous data, and they are different from the statistics that the Ministry of Health, Labour, and Welfare made and published.

The data of “Comprehensive survey of the living conditions” in Japan from 2001 to 2013 were used. To assess the status of households and income across Japan, the “Comprehensive survey of the living conditions” is conducted every year. A survey for health status is also conducted every 3 years (Ministry of Health, Labour and Welfare of Japan, 2020). The target districts of the survey are determined by stratified random sampling from all over Japan, and all households in the districts are subject to the survey. Participants respond to questionnaires asking about their income, household, health, savings, and care status. The number of responses (response rate) for each of the analyzed years are as follows: 31,871 households (79.5%) in 2001, 25,621 (70.1%) in 2004, 24,578 households (67.7%) in 2007, 27,225 households (75.7%) in 2010, and 27,081 households (74.4%) in 2013 (Ministry of Health, Labour and Welfare of Japan, 2020). We used anonymous data from the survey, which are randomly sampled from the aggregated households. Households that could be identified, such as those with a large number of household members, were eliminated in advance by the Ministry of Health, Labour, and Welfare. We obtained permission from the Ministry of Health, Labour, and Welfare to use the data. The anonymous data were also used in several previous studies (Fukuda et al., 2007; Fukuda et al., 2011; Wada et al., 2015; Fujiwara et al., 2018; Kaso et al., 2019; Maeda et al., 2020).

*Outcome and Explanatory Variables*

Participation in breast cancer screening was used as the outcome. Participants were asked whether they participated in a breast cancer screening in the past year. We used age group, marital status, living arrangements, education level, household income, employment status, smoking status, regular outpatient visit status, and self-rated health status as explanatory variables because these were shown to be predictors of cancer screening in previous studies. Age groups from 0 to 79 years by 5-year increments were available in the data. Then, we aggregated the age groups into age groups by 10-year increments. Women between the ages of 40 and 69 were included in our analysis.

Four types of marital status (married, never-married, widowed, and divorced) were available in the data. We aggregated widowed and divorced into a single group because the number of widowed women was small. Data on the number of household members were available. Using this, we created a binary variable for living arrangements based on whether the individual was living with others or not.

Six educational levels were available, including elementary school or junior high school, high school, vocational school, junior college or technical college, university, and graduate school. We aggregated these educational levels into 3 levels: low (elementary school or junior high school), middle (high school and vocational school), and high (junior college, technical college, university, and graduate school). Individuals whose educational level was unknown were treated as “unknown” for the educational level. Educational level for the subjects was available for 2010 and 2013.

The quantile of the household income was calculated, and individuals were grouped into 4 groups based on household income level. To determine employment status, each individual was grouped based on the company size of their workplace. 6 employment statuses existed: working for a large scale company (> 1000 employees or government or municipal offices), working for a middle scale company (30–1000 employees), working for a small scale company (< 30 employees), self-employed, other (fixed-time workers, company officer, etc.), and unemployed.

Individuals who smoke every day or sometimes were categorized as smokers, and individuals who do not smoke or who are former smokers were categorized as non-smokers. Also, individuals were divided based on whether or not they regularly visited an outpatient service. Moreover, the 5-grade scale used for self-rated health status was grouped into 3 statuses for our analysis: good (good or generally good), normal (normal), and bad (not good or bad).

*Statistical Analysis*

Individuals whose working status, smoking status, outpatient visit status, or self-rated health status were uncertain were removed from the analysis. First, baseline characteristics of the data were tallied for each of the analyzed years. The participation rate for cancer screening and its 95% confidence interval was calculated for each of the factors for each year. Then, to analyze the association between each factor and the participation rate, the annual percentage change of the participation rate by each predictor was calculated. Finally, multivariate logistic regression analysis was conducted. The data from 2010 and 2013 were used for logistic analysis because educational level was available for these years. All statistical analysis was conducted using R3.6.3 (https://www.R-project.org/).

## Results

**Figure 1:**
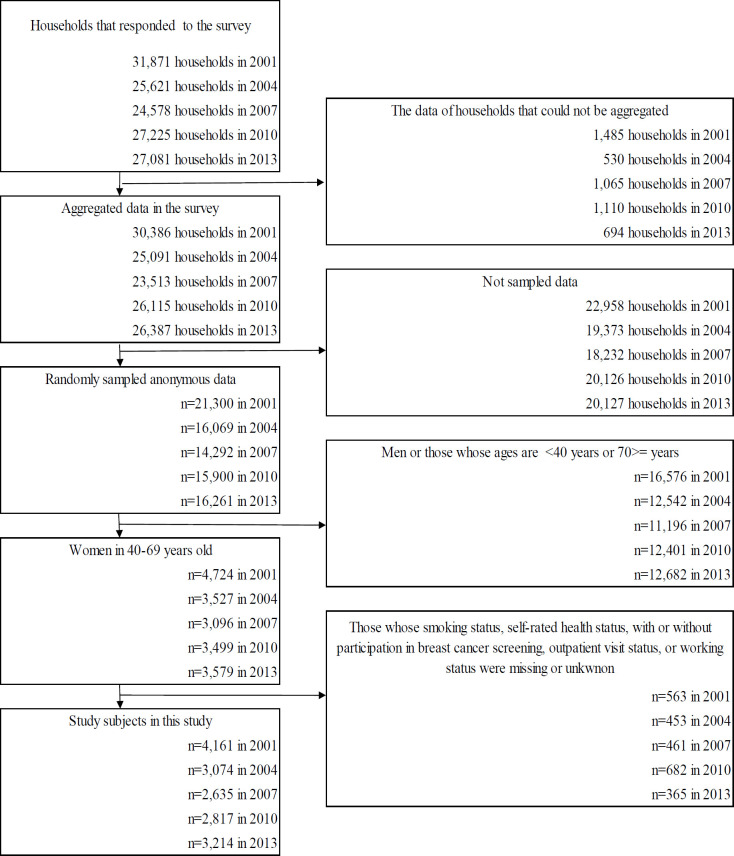
Flowchart for Selecting Study Subjects

Figure 1 shows the flowchart for selecting study subjects. Table 1 shows the basic characteristics of the study subjects for each year. The rate of participation for breast cancer screening increased throughout the analyzed years.

Table 2 shows the participation rate of breast cancer screening and its 95% confidence interval by predictor for each year, as well as the average annual percentage change. The participation rate increased from 2001 to 2013, regardless of the status of the predictors. The degree of increase of the participation rate in the 40s age group was evident during the analyzed periods compared with the 60s age group. Although the participation rate of married women was the largest based on marital status, the degree of increase in participation was the largest in never-married women. Obvious hierarchical relationships also existed in the participation rate among educational level and household income quantiles. A hierarchical relationship also existed in the participation rate based on scale of the workplace; the participation rates for self-employed and unemployed women were particularly low. Moreover, the participation rate of smokers was significantly lower than that of non-smokers throughout the analyzed periods.

Table 3 shows the results of multivariate logistic regression for the predictors of participation rate in breast cancer screening using data from 2010 and 2013. A significant association with the screening rate was observed for age, marital status, educational level, household income, employment status, smoking status, outpatient visit status, self-rated health status, and year.

**Table 1:** Basic Characteristics of the Study Subjects in Each Year

	2001 n=4161 N(%)	2004 n=3074 N(%)	2007 n=2635 N(%)	2010 n=2817 N(%)	2013 n=3214 N(%)
Age					
40-49 years	1321 (31.7)	929 (30.2)	818 (31.0)	916 (32.5)	1018 (31.7)
50-59 years	1581 (38.0)	1146 (37.3)	951 (36.1)	905 (32.1)	998 (31.1)
60-69 years	1259 (30.3)	999 (32.5)	866 (32.9)	996 (35.4)	1198 (37.3)
Marital status					
Married	3383 (81.3)	2510 (81.7)	2178 (82.7)	2313 (82.1)	2532 (78.8)
Never-married	217 (5.2)	146 (4.7)	128 (4.9)	176 (6.2)	246 (7.7)
Divorced/Widowed	561 (13.5)	418 (13.6)	329 (12.5)	328 (11.6)	436 (13.6)
Living arrangements					
Living with others	3862 (92.8)	2856 (92.9)	2466 (93.6)	2634 (93.5)	2978 (92.7)
Living alone	299 (7.2)	218 (7.1)	169 (6.4)	183 (6.5)	236 (7.3)
Educational level					
High				687 (24.4)	801 (24.9)
Middle				1571 (55.8)	1858 (57.8)
Low				314 (11.1)	287 (8.9)
Unknown				245 (8.7)	268 (8.3)
Household income					
4^th ^(highest) quantile	1118 (26.9)	820 (26.7)	746 (28.3)	794 (28.2)	898 (27.9)
3^rd^ quantile	1051 (25.3)	783 (25.5)	687 (26.1)	745 (26.4)	794 (24.7)
2^nd^ quantile	969 (23.3)	726 (23.6)	622 (23.6)	648 (23.0)	728 (22.7)
1^st^ (lowest) quantile	1023 (24.6)	745 (24.2)	580 (22.0)	630 (22.4)	794 (24.7)
Employment status					
Large scale company	241 (5.8)	174 (5.7)	138 (5.2)	205 (7.3)	240 (7.5)
Middle scale company	522 (12.5)	374 (12.2)	306 (11.6)	409 (14.5)	521 (16.2)
Small scale company	492 (11.8)	317 (10.3)	237 (9.0)	288 (10.2)	344 (10.7)
Self-employed	576 (13.8)	404 (13.1)	361 (13.7)	320 (11.4)	275 (8.6)
Others	629 (15.1)	472 (15.4)	473 (18.0)	421 (14.9)	550 (17.1)
Unemployed	1701 (40.9)	1333 (43.4)	1120 (42.5)	1174 (41.7)	1284 (40.0)
Smoking status					
Non-smoker	3627 (87.2)	2706 (88.0)	2334 (88.6)	2501 (88.8)	2863 (89.1)
Smoker	534 (12.8)	368 (12.0)	301 (11.4)	316 (11.2)	351 (10.9)
Outpatient visit status					
Yes	1781 (42.8)	1290 (42.0)	1138 (43.2)	1306 (46.4)	1448 (45.1)
No	2380 (57.2)	1784 (58.0)	1497 (56.8)	1511 (53.6)	1766 (54.9)
Self-rated health status					
Good	1471 (35.4)	1135 (36.9)	838 (31.8)	912 (32.4)	1076 (33.5)
Normal	2081 (50.0)	1482 (48.2)	1402 (53.2)	1492 (53.0)	1736 (54.0)
Bad	609 (14.6)	457 (14.9)	395 (15.0)	413 (14.7)	402 (12.5)
Breast cancer screening					
Nor participated	3161 (76.0)	2265 (73.7)	1914 (72.6)	1898 (67.4)	1950 (60.7)
Participated	1000 (24.0)	809 (26.3)	721 (27.4)	919 (32.6)	1264 (39.3)

**Table 2:** Participation Rate of Breast Cancer Screening and Its 95% Confidence Interval by Predictors for Each Year, and Its Average Annual Percentage Change

	2001	2004	2007	2010	2013	AAPC
	Rate (95% CI)	Rate (95% CI)	Rate (95% CI)	Rate (95% CI)	Rate (95% CI)	
Age						
40-49 years	22.7 (20.5-25.0)	28.1 (25.2-31.0)	26.9 (23.9-29.9)	36.7 (33.6-39.8)	43.8 (40.8-46.9)	7.75
50-59 years	26.5 (24.3-28.7)	28.3 (25.7-30.9)	30.1 (27.2-33.0)	34.4 (31.3-37.5)	43.0 (39.9-46.1)	5.19
60-69 years	22.3 (20.0-24.6)	22.4 (19.8-25.0)	24.8 (21.9-27.7)	27.3 (24.5-30.1)	32.5 (29.8-35.1)	3.81
Marital status						
Married	25.7 (24.2-27.2)	27.4 (25.6-29.1)	28.6 (26.7-30.5)	33.6 (31.7-35.6)	41.5 (39.6-43.4)	5.12
Never-married	14.7 (10.0-19.5)	19.2 (12.8-25.6)	18.8 (12.0-25.5)	31.2 (24.4-38.1)	31.3 (25.5-37.1)	9.41
Divorced/Widowed	17.6 (14.5-20.8)	22.5 (18.5-26.5)	22.8 (18.3-27.3)	26.2 (21.5-31.0)	31.4 (27.1-35.8)	6.53
Living arrangements						
Living with others	24.6 (23.2-26.0)	26.9 (25.3-28.5)	27.8 (26.0-29.6)	33.0 (31.2-34.7)	40.0 (38.3-41.8)	5.22
Living alone	16.7 (12.5-21.0)	18.8 (13.6-24.0)	20.7 (14.6-26.8)	27.9 (21.4-34.4)	30.5 (24.6-36.4)	6.89
Educational level						
High				41.9 (38.2-45.6)	50.6 (47.1-54.0)	6.92
Middle				31.0 (28.7-33.3)	37.2 (35.0-39.4)	6.67
Low				21.0 (16.5-25.5)	23.3 (18.5-28.2)	3.65
Unknown				31.8 (26.0-37.7)	37.3 (31.5-43.1)	5.77
Household income						
4^th^ (highest) quantile	31.1 (28.4-33.8)	32.9 (29.7-36.1)	32.4 (29.1-35.8)	40.1 (36.6-43.5)	49.9 (46.6-53.2)	5.04
3^rd^ quantile	23.3 (20.8-25.9)	26.7 (23.6-29.8)	31.7 (28.3-35.2)	34.8 (31.3-38.2)	41.4 (38.0-44.9)	6.47
2^nd^ quantile	21.8 (19.2-24.4)	24.4 (21.3-27.5)	22.8 (19.5-26.1)	27.6 (24.2-31.1)	34.6 (31.2-38.1)	4.89
1^st^ (lowest) quantile	19.2 (16.7-21.6)	20.5 (17.6-23.4)	20.5 (17.2-23.8)	25.9 (22.5-29.3)	29.6 (26.4-32.8)	4.51
Employment status						
Large scale company	37.8 (31.6-43.9)	42.0 (34.6-49.3)	38.4 (30.3-46.5)	49.8 (42.9-56.6)	56.7 (50.4-62.9)	4.17
Middle scale company	25.5 (21.7-29.2)	25.9 (21.5-30.4)	28.8 (23.7-33.8)	37.4 (32.7-42.1)	44.5 (40.3-48.8)	6.21
Small scale company	22.2 (18.5-25.8)	32.2 (27.0-37.3)	26.2 (20.6-31.8)	32.3 (26.9-37.7)	44.5 (39.2-49.7)	8.37
Self-employed	21.7 (18.3-25.1)	22.5 (18.5-26.6)	26.3 (21.8-30.9)	24.7 (20.0-29.4)	34.5 (28.9-40.2)	4.92
Others	25.1 (21.7-28.5)	28.8 (24.7-32.9)	33.6 (29.4-37.9)	34.0 (29.4-38.5)	40.7 (36.6-44.8)	5.18
Unemployed	22.6 (20.6-24.6)	23.3 (21.0-25.5)	23.6 (21.1-26.1)	29.7 (27.1-32.3)	33.0 (30.4-35.6)	3.83
Smoking status						
Non-smoker	25.5 (24.1-26.9)	27.3 (25.6-29.0)	28.6 (26.7-30.4)	33.9 (32.1-35.8)	40.9 (39.1-42.7)	5.03
Smoker	14.2 (11.3-17.2)	19.0 (15.0-23.0)	17.9 (13.6-22.3)	22.2 (17.6-26.7)	26.5 (21.9-31.1)	7.22
Outpatient visit status						
Yes	27.8 (25.7-29.9)	28.9 (26.4-31.4)	30.8 (28.1-33.4)	34.4 (31.8-37.0)	41.2 (38.7-43.8)	4.02
No	21.2 (19.6-22.9)	24.4 (22.4-26.4)	24.8 (22.6-27.0)	31.1 (28.8-33.4)	37.8 (35.5-40.0)	6.53
Self-rated health status						
Good	23.7 (21.5-25.8)	26.6 (24.0-29.2)	28.0 (25.0-31.1)	35.5 (32.4-38.6)	42.9 (40.0-45.9)	6.75
Normal	24.6 (22.8-26.5)	26.5 (24.3-28.8)	27.2 (24.8-29.5)	31.4 (29.0-33.7)	38.7 (36.4-41.0)	4.78
Bad	23.0 (19.6-26.3)	24.9 (21.0-28.9)	26.6 (22.2-30.9)	30.8 (26.3-35.2)	32.3 (27.8-36.9)	3.37

CI, Confidence interval; AAPC, average annual percentage change

**Table 3:** Results of Multivariate Logistic Regression for the Predictors of Participation Rate of Breast Cancer Screening Using the Data of 2010 And 2013

	Adjusted OR (95% CI)	
Age		
40-49 years	1.00	
50-59 years	0.90 (0.79, 1.04)	
60-69 years	0.77 (0.66, 0.90)	***
Marital status		
Married	1.00	
Never-married	0.66 (0.52, 0.84)	***
Divorced/Widowed	0.81 (0.66, 0.98)	*
Living arrangements		
Living with others	1.00	
Living alone	1.13 (0.87, 1.48)	
Educational level		
High	1.00	
Middle	0.71 (0.62, 0.80)	***
Low	0.49 (0.39, 0.62)	***
Unknown	0.75 (0.60, 0.93)	**
Household income		
4^th^ (highest) quantile	1.00	
3^rd^ quantile	0.84 (0.73, 0.98)	*
2^nd^ quantile	0.69 (0.59, 0.81)	***
1^st ^(lowest) quantile	0.64 (0.54, 0.77)	***
Employment status		
Large scale company	1.00	
Middle scale company	0.73 (0.57, 0.92)	**
Small scale company	0.66 (0.51, 0.85)	**
Self-employed	0.45 (0.35, 0.59)	***
Other	0.62 (0.49, 0.78)	***
Unemployed	0.49 (0.40, 0.61)	***
Smoking status		
Non-smoker	1.00	
Smoker	0.56 (0.46, 0.68)	***
Outpatient visit status		
Yes	1.00	
No	0.68 (0.60, 0.76)	***
Self-rated health status		
Good	1.00	
Normal	0.86 (0.76, 0.97)	*
Bad	0.70 (0.58, 0.85)	***
Year		
2010	1.00	
2013	1.35 (1.21, 1.51)	***

*p<0.05; ** p<0.01; *** p<0.001; OR, Odds ratio; CI, Confidence interval

## Discussion

We analyzed the trend of participation rate in breast screening by its possible predictors and identified some predictors using recent nationally representative survey data. As a result, we found that a disparity existed in the participation rate by marital status, income, and employment status throughout the analyzed periods. Also, we determined that educational level was related to the participation rate. We discuss the association of each predictor and the breast cancer screening.

Regarding the trend of the participation rate, an increase in the participation rate was observed in all predictor groups used in this study, whereas it was known that the participation rate in breast cancer screening was increasing in all of Japan (Ministry of Health, Labour and Welfare of Japan, 2020). Moreover, we found that the degree of increase became larger from 2007 to 2013 compared with the period from 2001 to 2007. The Basic Plan to Promote Cancer Control Programs and the Cancer Control Act was launched in 2007 (Saito, 2012). After this, an increase in participation in cancer screening was advocated across the whole country. Therefore, a possibility exists that each municipality and workplace in Japan increased their focus on the recommendation for cancer screening more than before. The increase in participation in breast cancer screening was evident in the 40s age group. Until 2003, the target age for breast cancer screening was more than 50 years old in Japan. However, in 2003, the age was lowered thereafter to women older than 40 years old (Tsunoda, 2017), which might also have affected our results. Furthermore, our analysis showed that the degree of increase in the participation rate was particularly evident among never-married individuals during the periods analyzed. The association between marital status and the participation rate or other health behaviors was shown in previous studies (El-Haddad et al., 2015; Hanske et al., 2016), and increased social support among married individuals is believed to be a reason for this phenomenon. The rate of cohabitation with parents for never-married individuals increased in the analyzed periods (Ministry of Internal Affairs and Communications, 2020), which might be related to the phenomenon.

The association between participation rate and socioeconomic factors (educational level, household income, scale of workplace) were shown, and lower educational level was significantly associated with a lower participation rate. Although the association between participation and educational level has been shown for screening rates of other types of cancer (Kaso et al., 2019; Maeda et al., 2020), the association for breast cancer has not yet been verified by a study using nationally representative data in Japan. A possible reason for the association between participation in cancer screening and educational level is that women with lower educational level tend to exhibit poorer health literacy (Furuya et al., 2015; Kaso et al., 2019). Therefore, it may be that women with lower education do not possess enough knowledge about breast cancer screening or breast cancer and may not perceive the importance of the screening.

Household income was also associated with the participation rate in this study. Individuals with lower household income tended to demonstrate lower screening participation. Although it has been shown that household income was associated with the participation rate in a previous study using data from 2001 (Fukuda et al., 2005), the association was also present in more recent years. Additionally, the association was found to be evident after adjusting other predictors such as educational level or smoking prevalence. In Japan, participation in cancer screening is not free of charge, and even cancer screening conducted by the municipality incurs a small charge. Therefore, it is believed that some individuals do not participate in breast cancer screening for financial reasons.

Also, individuals working for larger companies demonstrated a higher participation rate, whereas participation rates for self-employed and unemployed individuals were particularly low. The association between participation rate in lung cancer screening and company size was also shown in a previous study (Maeda et al., 2020). In Japan, a large number of people participate in cancer screenings conducted by their workplace or insurer (Ministry of Health, Labour and Welfare of Japan, 2020) and larger companies with more employees tend to conduct cancer screenings more than smaller companies. Conversely, self-employed or unemployed individuals exhibit no opportunities to participate in cancer screenings conducted by the workplace, which likely contributes to their lower participation rate.

Smoking prevalence and self-rated health status were also associated with participation rate in this study. The association between smoking status and participation was also indicated in other countries (Lee et al., 2010; Sanford et al., 2019), although the reason is uncertain. Psychological attitudes toward health behavior is one possible factor for this phenomenon (Lee et al., 2010), with the possibility of common psychological attitudes or states related to these risky health behaviors. Self-rated health is known to be associated with low socioeconomic status (SES) or risky health behavior (Wang et al., 2012; Wada et al., 2015; Hanibuchi et al., 2016). It is also known that self-rated health medicates SES and participation in colorectal cancer screening in another country (Miles et al., 2011).

Although it is known that socioeconomic disparity existed regarding breast cancer screening, we found that the socioeconomic disparity did not decrease over the years and still existed in more recent years. First, household income was associated with participation rate. In order to increase the participation rate among women with lower income, providing screenings to this group free of charge may be necessary, as is done in Korea (Goto et al., 2015). Second, the rates of participation for unemployed and self-employed individuals were particularly low. Each municipality must seek methods to recommend cancer screening for those individuals. Low SES was shown to be associated with low health literacy (Furuya et al., 2015), and further efforts to explain the importance of screening to individuals with low SES are needed. Conversely, high SES was shown to be associated with higher breast cancer mortality rates in Japan because of reasons such as reproductive factors and alcohol consumption (Fujino et al., 2008; Zaitsu et al., 2018). However, the disparity in participation in breast cancer screening could affect the relationship between SES and the breast cancer mortality rate in the future. Also, breast cancer screening provides not only benefits (Myers et al., 2015), but also harm. The possibility exists that some women who do not participate in the screening because of this risk of harm. In other countries, low SES was shown to be associated with fatalistic cancer beliefs (Assari et al., 2019). A possibility exists that the association is also present in Japan because women with low household income may not be able to afford the treatment fee, even if breast cancer is detected in the screening. Therefore, collecting data regarding reasons for non-participation in the national survey in the future will be meaningful. 

Some limitations are present in this study. We analyzed data from self-reported questionnaires, and some inaccuracy could be present in the responses. There was also some missing information in the variables used in this study, which might have affected our results. A possibility exists that the screening participation rate was actually lower among individuals whose participation status was unknown. Moreover, the latest data analyzed were from 2013. We had to acquire anonymous data from the Ministry of Health, Labour and Welfare of Japan to conduct this study. Although the latest year in which the national survey was conducted is 2019, the latest anonymous data the Ministry of Health, Labour and Welfare of Japan is providing at the moment is those of 2013; thus, we could analyze only the anonymous data until 2013 for this study. An analysis using more recent data should be conducted in the future. Conversely, the analyzed data were from a nationally representative survey, and the results of this study are considered to be generalizable to all of Japan.

In summary, we found that non-married women, women with lower educational level, women with low household income, self-employed or unemployed women, smokers, and women with low self-rated health status were significantly less likely to participate in breast cancer screening in Japan. In addition, significant differences were found regarding participation rate in breast cancer screening depending on marital status, household income, employment status, and smoking status throughout the analyzed years. Therefore, further recommendations for breast cancer screening are needed, particularly directed toward women of lower SES and those who as self-employed or unemployed to increase the rate of participation in Japan.
